# The oxidative potential of particulate matter (PM) in different regions around the world and its relation to air pollution sources†

**DOI:** 10.1039/d2ea00043a

**Published:** 2022-07-05

**Authors:** Vahid Jalali Farahani, Abdulmalik Altuwayjiri, Milad Pirhadi, Vishal Verma, Ario Alberto Ruprecht, Evangelia Diapouli, Konstantinos Eleftheriadis, Constantinos Sioutas

**Affiliations:** University of Southern California, Department of Civil and Environmental Engineering 3620 S. Vermont Ave, KAP210 Los Angeles California 90089 USA sioutas@usc.edu +1-213-744-1426 +1-213-740-6134; California Air Resources Board Sacramento California USA; University of Illinois at Urbana Champaign, Department of Civil and Environmental Engineering Urbana Illinois USA; International Society of Doctors for Environment (ISDE) Italy; Environmental Radioactivity Laboratory, N.C.S.R. Demokritos 15341 Attiki Greece; Majmaah University, Department of Civil and Environmental Engineering Majmaah Riyadh Saudi Arabia

## Abstract

In this study, we investigated the impact of urban emission sources on the chemical composition of ambient particulate matter (PM) as well as the associated oxidative potential. We collected six sets of PM samples in five urban location sites around the world over long time periods varying from weeks to months, intentionally selected for their PM to be dominated by unique emission sources: (1) PM_2.5_ produced mainly by traffic emissions in central Los Angeles, United States (US); (2) PM_2.5_ dominated by biomass burning in Milan, Italy; (3) PM_2.5_ formed by secondary photochemical reactions thus dominated by secondary aerosols in Athens, Greece; (4) PM_10_ emitted by refinery and dust resuspension in Riyadh, Saudi Arabia (SA); (5) PM_10_ generated by dust storms in Riyadh, SA, and (6) PM_2.5_ produced mainly by industrial and traffic emissions in Beirut, Lebanon. The PM samples were chemically analyzed and their oxidative potential were quantified by employing the dithiothreitol (DTT) assay. Our results revealed that the Milan samples were rich in water soluble organic carbon (WSOC) and PAHs, even exceeding the levels measured on Los Angeles (LA) freeways. The PM in Athens was characterized by high concentrations of inorganic ions, specifically sulfate which was the highest of all PM samples. The ambient PM in LA was impacted by the traffic-emitted primary organic and elemental carbon. Furthermore, the contribution of metals and elements per mass of PM in Riyadh and Beirut samples were more pronounced relative to other sampling areas. The highest intrinsic PM redox activity was observed for PM with the highest WSOC fraction, including Milan (biomass burning) and Athens (secondary organic aerosols, SOA). PM in areas characterized by high metal emissions including dust events, refinery and industry, such as Riyadh and Beirut, had the lowest oxidative potential as assessed by the DTT assay. The results of this study illustrate the impact of major emission sources in urban areas on the redox activity and oxidative potential of ambient PM, providing useful information for developing efficient air pollution control and mitigation policies in polluted areas around the globe.

## Abstract 2

Biomass burning and secondary organic aerosols formed by photochemical reactions lead to the highest oxidative potential among urban emission sources and therefore are one of the most hazardous sources in terms of ambient PM toxicity.

## Graphical abstract

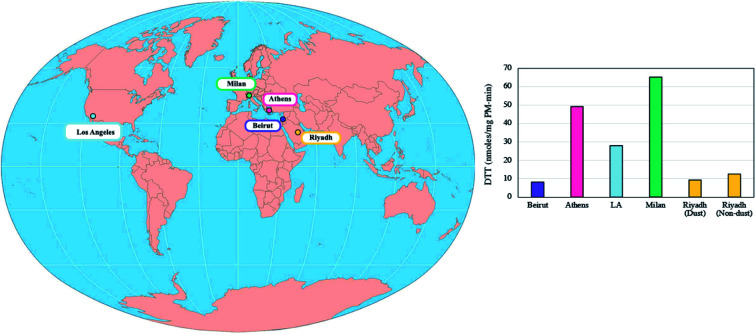

**Environmental significance**

Particulate matter (PM) is one of the main atmospheric drivers for adverse health effects on living organisms. However, the potential toxicity of ambient PM is not statistic variable, but rather a dynamic one and a function of its chemical composition and the emission source. In this study, we evaluated the chemical composition and the corresponding oxidative potential of ambient PM in various metropolitan areas, providing useful information for the impact of the urban emission source on the redox activity of ambient PM. This information can be utilized to develop efficient air pollution control policies in urban areas around the globe.

## 1. Introduction

Exposure to particulate matter (PM) has been associated with millions of premature deaths each year worldwide.^1–3^ According to numerous studies, particulate matter is responsible for a multitude of adverse health risks, including cardiovascular disorders,^4–7^ impaired mental health,^8–10^ carcinogenic diseases and respiratory problems.^11–15^ Due to the considerable differences in chemical composition of ambient particles formed from different emission sources, the PM mass concentration is probably not an ideal measure for the toxicity of PM. Therefore, researchers have proposed oxidative potential (OP), the capacity of aerosols to induce oxidative damage, as one of the metrics to reflect the acute and chronic health effects of PM exposure.^16–22^ The OP is generally measured by means of cellular and acellular (chemical) assays. One of the most commonly employed acellular methods is the dithiothreitol (DTT) assay. While DTT may not be a direct indicator for PM toxicity, it is a suitable proxy for redox activity and has been extensively employed in the literature.^23–26^ In this method, DTT is oxidized to its disulfide form after its interaction with redox-active chemicals in PM, and the linear decay rate of DTT is used as an index of the oxidative capacity of the PM.^27^ Numerous epidemiological and toxicological studies have been conducted to identify the PM components inducing oxidative activity.^16,28–33^ A recently published study by Bates *et al.*^16^ underscored the significant role of water-soluble total carbon (WSTC) in PM_10_ oxidative properties in the Alpine area. Lovett *et al.*^34^ observed significant associations between EC and OC (tracers of tailpipe emissions), WSOC (tracer of SOA), and heavy metals such as Ni, Cu, Zn, As, V, Cd, and Pb (tracers of non-tailpipe emissions) with the oxidative potential of ambient particulate matter in Beirut. Saffari *et al.*^24^ integrated the results of several studies that were conducted at different locations across the world and linked PM-induced oxidative potential with particle chemical composition. The authors of that study employed a cell-based microphage assay (*i.e.*, 2′,7′-dichlorodihydrofluorescein diacetate, DCFH-DA) to estimate the oxidative potential of PM. The results of that study included the PM collected in various areas globally almost a decade ago. However there has been substantial alteration in the emission sources of ambient PM in the studied locations such as Los Angeles, due to promulgation of vehicle and industrial emissions control technologies;^35^ in Italy, due to elevated domestic biomass burning;^36^ in Greece, due to reduction in fossil fuel use as well as the growing use of diesel passenger cars following the withdrawal of the ban on these types of vehicles;^37,38^ and in Lebanon, due to change in vehicle fleet composition and enhanced fuel consumption of diesel generators.^39,40^ To the best of our knowledge, no recent studies have evaluated the ambient PM chemical and potential toxicological characteristics in various densely populated cities with unique urban settings and emission sources.

The main objective of this study is to investigate the impact of different urban source emissions on the chemical and potential toxicological properties of ambient particulate matter (PM) by comparing the oxidative potential and chemical constituents of PM samples in various distinct locations throughout the globe, each dominated by a specific emission source. A series of comprehensive ambient air sampling campaigns were conducted in the metropolitan areas of Los Angeles (USA), Athens (Greece), Milan (Italy), Beirut (Lebanon), and Riyadh (Saudi Arabia). PM_2.5_ (particulate matter with aerodynamic diameters below 2.5 μm) and PM_10_ (particulate matter with aerodynamic diameters below 10 μm) collected on filters were analyzed for their carbonaceous content and chemical components (*i.e.*, water-soluble ions, metals, and trace elements). The DTT *in vitro* assay was deployed to determine the oxidative potential of these samples. Findings of this work advance our knowledge of complex source emission impacts on the PM oxidative potential and chemical composition in different environments and provide important insights for more targeted and cost-effective air pollution strategies in polluted areas around the globe.

## 2. Methodology

### 2.1. Sampling information

Six batches of PM samples with varied particle size fractions (*i.e.* PM_2.5_ or PM_10_) and sampling periods were the focus of this synthesis study. The PM samples were collected in cities of Los Angeles, Milan, Athens, Riyadh, and Beirut, each corresponding to unique set of PM emission sources. Table 1 summarizes the relevant information for each sampling batch. The samples in Riyadh were collected during two periods, one corresponding to dust storm events to measure the impact of dust particles on the oxidative activities and the other corresponding to a non-dust period to investigate the contribution of refinery emissions and dust resuspension to the PM oxidative potential. The samples in Athens were collected during the summertime when the rate of photochemical oxidation is at peak, enhancing the content of secondary organic aerosols (SOA) as the main PM emission source in that region. Furthermore, the high temperature in the summer minimizes the contribution of primary volatile and semi-volatile compounds to PM mass. Biomass burning is another major source generating PM redox activity in the metropolitan areas in the colder time period of the year. We investigated the impact of this emission source on the PM oxidative potential by incorporating the samples collected in Milan during the winter. According to our previous investigation, the overall oxidative potential of Milan's ambient particles during cold periods is largely induced by the biomass burning activities in this urban region.^41^ The ambient PM in cities of Los Angeles and Beirut are both heavily impacted by the traffic emissions. However, they are dissimilar in their PM chemical composition. The Beirut PM redox activity is dominated by the transition metals, compared to the LA area, in which organic compounds are the driving components in generating redox activity.^28^ Furthermore, particles in Beirut are also influenced by industrial emissions to a much higher degree than those in Los Angeles.

**Table 1:** Summary of information pertaining to the collected PM_2.5_ and PM_10_ (particulate matters with diameters below 2.5 and 10 μm, respectively) across different locations worldwide

City	Particle size	Dominant PM emission source	Sampling period(s)	Sampling days	Study
Los Angeles, USA	PM_2.5_	Traffic emissions	August 2018	24	Pirhadi *et al.*^85^
			Dec 2018 to Jan 2019		
Milan, Italy	PM_2.5_	Biomass burning	Dec 2018 to Feb 2019	14	Hakimzadeh *et al.*^41^
Athens, Greece	PM_2.5_	Secondary organic aerosols (SOA)	Aug 2020	20	Current study
Beirut, Lebanon	PM_2.5_	Traffic and industrial emissions	March 2020 and May 2020	34	Current study
Riyadh, Saudi Arabia	PM_10_	Dust and refinery emissions	December 2019 to August 2020	63	Altuwayjiri *et al.*^86^

### 2.2. Chemical analysis

The PM samples were chemically analyzed for their content of elemental carbon (EC), organic carbon (OC), water-soluble inorganic ions, polycyclic aromatic hydrocarbons (PAHs) as well as metals and trace elements by the Wisconsin State Lab of Hygiene (WSLH). The EC/OC content in the PM samples were measured *via* the Thermal Evolution/Optical Transmittance (TOT) analytic method using a model-4-semi-continuous OC/EC field analyzer (Sunset Laboratory Inc, USA).^42^ A small fraction of the filter was punched and kept in an oven, which was set at a temperature of 820 °C to capture the oxidized OC fraction. The oven temperature was decreased and then raised to the temperature of 860 °C through the mixture of helium and oxygen gases to measure the oxidized EC fraction. The WSOC fraction of PM samples was quantified through the extraction of PM in ultrapure water and the subsequent filtration (0.22 μm pore size) of the samples using a Sievers 900 total organic carbon (TOC) analyzer (GE Analytical Instruments, Boulder, CO, USA).^43^ The analysis of PM-bound metals and trace elements was performed by means of inductively coupled plasma-mass spectroscopy (ICPMS).^44,45^ A punch of the filter was digested in a mixture of nitric acid, hydrochloric acid, and hydrofluoric acid (0.6 mL 16 N HNO_3_, 0.2 mL 12 N HCl, 0.1 mL 28 N HF) using an automated, temperature- and pressure-regulated, trace analysis microwave system (Milestone Ethos+). The PAH concentrations was measured through chromatography/mass spectrometry (GC/MS) analysis as described in Sánchez *et al.*^46^ The remaining part of the filters were extracted in a mixture of ultrapure water and ethanol by means of ion chromatography using a Dionex Model DX-500 Ion Chromatograph to quantify the PM inorganic ions content including ammonium (NH_4_^+^), nitrate (NO_3_^−^), and sulfate (SO_4_^2−^).

### 2.3. PM oxidative potential

Oxidative potential of the PM samples was determined by employing an *in vitro* DTT assay.^47,48^ The DTT is one of the most commonly used acellular assays to quantify PM OP.^23,49^ The OP measured by the DTT assay has shown a stronger association with wide range of adverse health effects compared to PM_2.5_ mass concertation^50–53^ and therefore is a suitable metric to represent the health effects associated with PM exposure. Furthermore, past studies have shown that the DTT-based OP is sensitive to chemical composition,^16,32,47,54^ changes seasonally^55–57^ and is extremely responsive to episodic events such as wildfires,^58^ haze^59^ and fireworks.^60^

The quantification of PM oxidative potential using this assay involves the incubating DTT with PM aqueous extracts, and the DTT depletion rate is measured, which is proportional to the generation rate of PM-driven ROS. The PM samples were stored in a freezer at a temperature of −20 °C and extracted into an aqueous media using high purity Milli-Q water. The extracts were incubated in the mixture of potassium phosphate (KPO_4_) buffer and DTT, and then the linear rate of DTT consumption was quantified. Further details of the DTT methodology are available elsewhere.^56^ The intrinsic and extrinsic DTT values were estimated by normalizing DTT depletion rate to PM mass and sampled air volume, respectively. The intrinsic DTT values are in units of nmoles per min per mg of PM and reflect the oxidative potential of PM per unit mass, while the extrinsic DTT values are in units of nmol per min per m^3^ of air and indicate the oxidative potential per unit volume of air of the aerosol component. The consistency of the measured DTT was evaluated by positive control as well as multiple field blanks, which were included in every sample queue. The standard chemical employed for positive control was 9,10-phenanthraquinone (PQ) based on the reported sensitivities.^23,49^ The concentration of the PQ was 0.2 mM in the reaction vial of DTT while the average rate of positive control was 1.867 ± 0.094 μM min^−1^ or 0.037 ± 0.039 pmol (min μg)^−1^.^61^

## 3. Results and discussion

### 3.1. Chemical composition of PM

The PM chemical constituents were investigated by organic carbon (OC) and the associated water-soluble organic carbon (WSOC), elemental carbon (EC), metal and trace elements as well as inorganic ions including chloride (Cl^−^), nitrate (NO_3_^−^), sulfate (SO_4_^2−^), sodium (Na^+^), ammonium (NH_4_^+^) and potassium (K^+^). The total metallic content was calculated as the sum of the crustal and trace elements. The crustal portion of the metal elements was quantified using the following equation which is based on the oxidized form of metal elements:^62,63^

1 C = 1.89Al + 1.21K + 1.43Fe + 1.40Ca + 1.66Mg + 1.70Ti + 2.14Si

Silicon (Si) is not determined in the ICP-MS analysis; thus, the concentration of this specie was calculated as 3.41 times of aluminum concentration (Al).^62^ The average mass fraction of individual species for PM sample sets at each location is shown in Table 2.

**Table 2:** The mass fraction of PM chemical components in the studied cities^a^

	Beirut	Athens	Los Angeles	Riyadh (dust)	Riyadh (non-dust)	Milan
PM constituents (μg mg^−1^ PM)						
EC	10.04	13.36	23.05	6.71	23.00	28.05
OC	90.27	241.64	241.12	34.62	76.55	217.76
WSOC	51.98	147.40	68.62	12.86	44.08	118.79
Cl^−^	0.13	1.09	—	4.56	6.23	9.74
NO_3_^−^	0.94	3.93	179.15	20.73	40.45	224.75
SO_4_^2−^	93.41	264.17	56.41	37.37	77.97	40.29
Na	3.26	15.18	—	4.30	7.07	3.03
NH_4_^+^	31.89	69.57	46.83	2.67	8.33	91.05
K^+^	2.17	5.79	—	2.07	2.87	11.85

Metals (ng mg^−1^ PM)						
Ca	58 088.44	44 540.28	19 023.33	134 400.06	160 764.66	10 754.79
Al	13 476.45	18 532.70	7729.16	58 536.25	40 047.85	5424.49
**Fe**	8540.81	18 755.25	7333.21	39 446.27	28 165.42	5973.15
Mg	17 613.01	8073.74	9885.59	28 090.01	18 773.74	4964.33
Zn	4211.02	1348.21	453.39	221.65	2157.01	618.18
Ba	537.62	451.12	478.53	430.75	538.88	214.18
**Cu**	801.49	281.73	262.63	84.59	218.36	241.26
Ti	849.11	989.80	481.08	3848.63	2806.80	119.48
**Mn**	1032.63	387.01	118.95	758.83	562.91	158.37
**Pb**	210.67	259.08	55.92	46.60	185.52	293.95
**Ni**	586.03	145.63	47.45	95.74	70.91	37.14
Sn	56.92	134.82	71.62	11.14	33.22	147.62
**Cr**	199.16	203.04	86.14	118.88	92.63	111.43
**V**	29.06	132.10	17.78	112.59	97.13	6.60
Li	31.34	8.46	15.91	33.14	20.91	4.17
**Cd**	5.67	6.57	1.22	1.01	3.11	4.84
Pd	0.65	0.56	2.34	1.09	0.62	4.52

a Redox-active metals are highlighted in the table.

The PM_2.5_ sample sets in Milan were dominated by water-soluble inorganic ions (380.71 μg mg^−1^ PM) and OC (217.76 μg mg^−1^ PM), followed by trace amounts of metals (119.57 μg mg^−1^ PM). High levels of water-soluble organic carbon (WSOC) (118.79 μg mg^−1^ PM), a tracer of biomass burning during the cold periods in the absence of photochemistry,^64–66^ were observed in Milan as a result of significant biomass burning in that city during the sampling period.^41^ Nitrate (NO_3_^−^) and ammonium (NH_4_^+^) constituted the highest fraction of PM among inorganic ions (224.75 and 91.05 μg mg^−1^ PM, respectively), which further corroborates the impact of biomass burning on PM levels. This is also consistent with Daellenbach *et al.*^3^ findings in Europe, attributing high SOA levels in winter to the oxidation of the anthropogenic precursors, mainly from biomass burning activities.

Similar to Milan, the ambient fine particles in Athens displayed high levels of OC (241.64 μg mg^−1^ PM) and water-soluble inorganic ions (359.74 μg mg^−1^ PM). Markers of secondary organic aerosols (*i.e.*, SO_4_^2−^ and NH_4_^+^) were among the highest of all PM batches and dominated Athens's water-soluble inorganic ions, underscoring the impact of SOA on ambient PM loadings in this region. Furthermore, the WSOC per mass value was 147.40 μg mg^−1^ PM, a significant increase from that of measured in Beirut (51.98 μg mg^−1^ PM) and Riyadh during dust storms (12.86 μg mg^−1^ PM) and non-dust periods (44.08 μg mg^−1^ PM). The samples in Riyadh exhibited significant loadings of PM-bound metals during both dust (299.63 μg mg^−1^ PM) and non-dust (300.78 μg mg^−1^ PM) periods. The chemical composition of ambient PM_2.5_ in Los Angeles was primarily composed of carbonaceous compounds. The quantified EC (23.05 μg mg^−1^ PM) and OC (241.12 μg mg^−1^ PM) were among the highest of the studied sites in this heavily trafficked region, while WSOC constituted relatively small fraction (68.6 μg mg^−1^ PM) of the total organic carbon. Nitrate was the dominant specie (179.15 μg mg^−1^ PM) among the water-soluble inorganic ions, followed by sulfate (56.41 μg mg^−1^ PM) and ammonium (46.83 μg mg^−1^ PM). Moderate loadings of total metals (160.14 μg mg^−1^ PM) were also observed in the LA basin which have been mostly associated with the traffic sources including resuspended dust and the vehicle abrasion.^35,67^

**Fig. 1:**
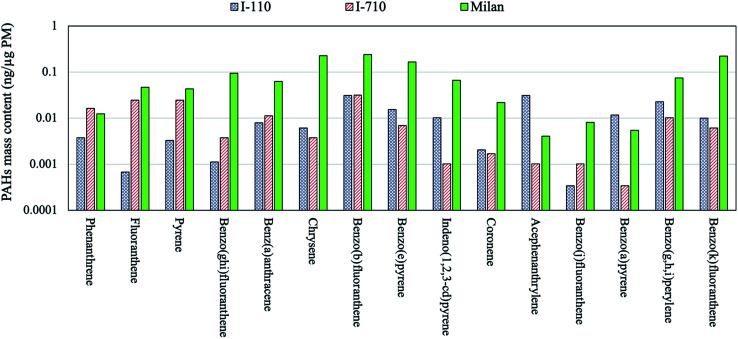
The comparison of PAH levels in Milan and Los Angeles I-110 and I-710 freeways.

Table 3 compares the PAH levels observed in Beirut, Athens, LA, Riyadh and Milan. The PAHs per PM mass concentration in Milan were substantially higher than other location sites across all PAH species. The PAHs are carcinogenic organic compounds which are typically released in particle phase during incomplete combustion from gasoline and diesel-fueled vehicles as well as biomass burning.^68–72^ The PAH content of the Milan samples, which are mainly driven by biomass burning as noted earlier, were significantly greater compared to the corresponding ambient levels in LA basin where PM is primary released from vehicle emissions. In fact, the observed values in Milan even exceeded our previously measured PAH values inside two major Los Angeles's freeways,^73^ as shown in Fig. 1. The cumulative PAH content in Milan were 1.35 ng μg^−1^ PM which is greater than that at I-110 (0.16 ng μg^−1^ PM) and I-710 (0.15 ng μg^−1^ PM) freeways by approximately one order of magnitude. These observations underscore the importance of biomass burning as one of the main driving factors in formation of PAHs. The low PAH levels in Athens during summer are probably the result of higher photo-degradation rate of these species with oxidizing gases (ozone, nitrogen oxides, hydrogen peroxide, *etc.*),^74,75^ as well as increased partitioning to the gas phase,^76^ due to the higher ambient temperatures in that time of year. Similarly, Riyadh and Beirut are both Middle Eastern cities associated with very high temperature events during spring and summer, resulting in minor PAH levels for the reasons noted earlier. Fewer number of PAH species were detected in the coastal city of Beirut due to the frequent daytime on-shore winds at this site, which increases the atmospheric dilution and coupled with the high temperature, it can enhance the volatilization, photodegradation and dispersion of PAHs.^28^

**Table 3:** The mass fraction of PAH components in the studied cities^a^

PAH species (ng μg^−1^ PM)	Beirut	Athens	LA	Riyadh (dust)	Riyadh (non-dust)	Milan
Phenanthrene	<LOD	0.0076	0.0030	0.0002	0.0042	0.0124
Fluoranthene	0.0010	<LOD	0.0026	0.0004	0.0075	0.0462
Pyrene	0.0008	<LOD	0.0031	0.0002	0.0070	0.0433
Benzo(*ghi*)fluoranthene	<LOD	<LOD	0.0005	0.0001	0.0048	0.0945
Benz(*a*)anthracene	<LOD	<LOD	0.0042	<LOD	<LOD	0.0623
Chrysene	<LOD	<LOD	0.0002	0.0001	0.0047	0.2253
Benzo(*b*)fluoranthene	<LOD	<LOD	0.0109	0.0007	0.0249	0.2402
Benzo(*k*)fluoranthene	<LOD	<LOD	0.0016	0.0001	0.0040	0.2214
Benzo(*j*)fluoranthene	<LOD	<LOD	<LOD	<LOD	<LOD	0.0080
Benzo(*e*)pyrene	<LOD	<LOD	0.0090	0.0006	0.0175	0.1665
Benzo(*a*)pyrene	<LOD	<LOD	<LOD	<LOD	<LOD	0.0054
Indeno(1,2,3-*cd*)pyrene	<LOD	<LOD	<LOD	<LOD	0.0144	0.0665
Benzo(*g*,*h*,*i*)perylene	0.0003	<LOD	0.0006	0.0002	0.0087	0.0743
Coronene	<LOD	<LOD	<LOD	<LOD	0.0072	0.0217
Dibenzo(*a*,*e*)pyrene	<LOD	<LOD	<LOD	<LOD	<LOD	0.0016

a The values below limits of detection (LOD) are shown as <LOD.

### 3.2. Comparison of PM oxidative potential among various locales

Table 4 shows the oxidative potential measured by the DTT assay normalized by PM mass (intrinsic DTT) as well as extrinsic DTT values. The data shown in Table 4 illustrate a wide range of oxidative responses measured by the DTT assay, underscoring the impact of location-specific emission sources and the resulting PM chemical composition on the PM oxidative stress. The lowest intrinsic level was measured in Beirut, where intrinsic DTT consumption rate was 8.22 ± 1.27 nmoles (min mg)^−1^. The PM sample sets collected in Athens and Milan exhibited 4–7 times higher DTT consumption rate, which in part can be attributed to the greater WSOC content of PM, originated from biomass burning and secondary aerosols, respectively in these cities. In agreement with this observation, Daellenbach *et al.*,^3^ who have investigated the sources of PM and oxidative potential across Europe, reported significant intrinsic oxidative potential from residential biomass burning activities. We will discuss the impact of different chemical constituents on the PM oxidative potential in detail in the following section. The intrinsic DTT values in Riyadh were somewhat higher for the PM collected during the non-dust period (12.53 ± 1.43 nmoles (min mg)^−1^) relative to those collected in a dust event period (9.32 ± 0.80 nmoles (min mg)^−1^). The local traffic emission sources in Los Angeles resulted in DTT activity of 28.10 ± 5.23 nmoles (min mg)^−1^. The extrinsic DTT consumption rates spanned from 0.35 ± 0.04 nmoles (min m^3^)^−1^ in LA to 5.62 ± 0.19 nmoles (min m^3^)^−1^ in Athens. The different trends in volume-normalized DTT values compared to the mass-normalized approach can be attributed to the higher PM mass concentration in the respective samples, resulting into greater exposure to redox-active aerosols despite the lower capability of PM chemical constituents in inducing oxidative activity.

**Table 4:** The intrinsic and extrinsic DTT activity for the collected PM batches across the globe

Location	PM size	Intrinsic DTT (nmoles (min mg)^−1^)	Extrinsic DTT (nmoles (min m^3^)^−1^)
Milan	PM_2.5_	65.29 ± 5.17	3.38 ± 0.26
Athens	PM_2.5_	49.20 ± 1.66	5.62 ± 0.19
Beirut	PM_2.5_	8.22 ± 1.27	3.51 ± 0.54
Los Angeles	PM_2.5_	28.10 ± 5.23	0.35 ± 0.04
Riyadh – dust period	PM_10_	9.32 ± 0.80	1.85 ± 0.15
Riyadh – non-dust period	PM_10_	12.53 ± 1.43	1.05 ± 0.12

### 3.3. The impact of emission sources on PM oxidative potential

**Fig. 2:**
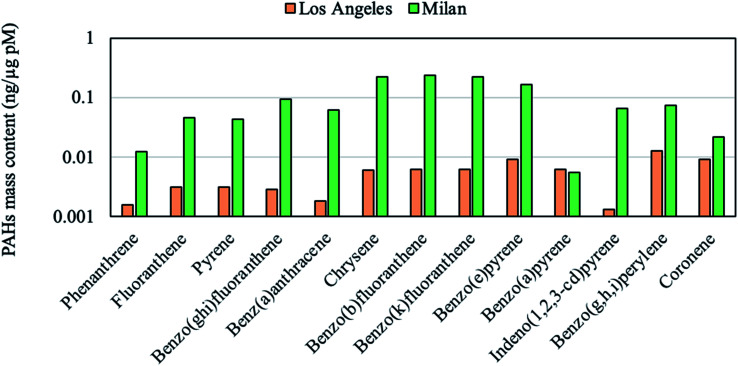
The comparison of PAH mass fractions (ng μg^−1^ of PM) in the ambient PM during and after a wildfire in LA to that of Milan.

We performed a linear regression analysis between measured PM chemical constituents and the DTT activities at different sites to identify the emission sources linked to PM oxidative potential, the results of which are summarized in Table 5. In order to perform the regression analysis, we employed the average values for the PM chemical constituents (shown in Tables 2 and 3) and intrinsic DTT rates (Table 4) for sample sets collected at individual locations. We then estimated the correlation coefficient and *p*-values based on the regression of the average values of DTT and chemical components at these studied sites. According to the table, the DTT activity is mostly correlated with the K^+^ (*R* = 0.94), a marker of biomass burning reported in the literature.^27,66,77^ The observed regression is also statistically significant (*p* = 0.06), corroborating the robust redox activity of this emission source. Strong and significant (*p* = 0.08) correlation between WSOC mass fraction and DTT activities (*R* = 0.89) was also observed. This observation agrees with previous studies reporting WSOC as one of the driving factors in the DTT consumption rate.^26,32^ The highest WSOC content was measured at Athens with the mass fraction of 147 μg mg^−1^ PM, followed by Milan (∼118 μg mg^−1^ PM), Beirut (∼52 μg mg^−1^ PM) and Riyadh during non-dust (∼44 μg mg^−1^ PM) and dust periods (∼13 μg mg^−1^ PM). WSOC is mainly formed by biomass burning and secondary photochemical reactions,^78–80^ however the formation of WSOC *via* photochemical reactions in winter-time is limited by the low temperatures. Thus, the large fraction of WSOC in Milan, which exhibited highest DTT activity among all locations, is most likely associated with the domestic biomass burning activities in this region. The water-insoluble organic carbon (WIOC) content, calculated as the difference between OC and WSOC, was also significantly correlated with the DTT activity (*R* = 0.71, *p* = 0.02). Since the DTT analysis was performed on the extracted filters, driven by water-soluble compounds, the high correlation of insoluble fraction of organic compounds with DTT values can be attributed to the fact that WIOC is likely emitted from similar sources as water-soluble DTT-active compounds. The PAHs have also clearly contributed to the overall redox activity of PM as corroborated by the regression values (*R* = 0.74, *p* = 0.03). PAH exposure can activate aryl hydrocarbon receptor (AhR) which causes the inflammation through inducing pro-inflammatory molecules and upregulation of pro-inflammatory genes.^81,82^ Biomass burning is one of the major sources of PAHs, as discussed in section 3.1. The significant PM loadings of total PAH in Milan were consistent with the high DTT consumption rate at this site. To illustrate the importance of different biomass burning types on PAH emissions, in Fig. 2 we compared the PAH levels and DTT values in Milan to the corresponding values in LA during a wildfire event.^58^ The PAH levels in Milan (79.5 ng mg^−1^ PM) were higher than that of LA (0.08 ng μg^−1^ PM) by almost two orders of magnitude. The substantial difference in PAH levels can be attributed to the different biomass burning sources in these two cities, where Milan's emissions are mostly from domestic heating whereas the LA's biomass burning emissions were dominated by a wildfire event. Furthermore, the content of the winter-time PAH of the PM samples in Milan was impacted by local meteorological conditions (temperature of 4 °C and relative humidity of 73%) and atmospheric stagnation, leading to higher PAH concentrations relative to corresponding values in LA, which were associated with higher temperature (26 °C) and lower relative humidity (31%) prevailing during the wildfire episodes. The higher PAH content in Milan was also consistent with the city's higher DTT activity (65.29 ± 5.17 nmoles (min mg)^−1^) compared to that of LA during wildfire (24.10 ± 8.12 nmoles (min mg)^−1^). This observation demonstrates that PM emitted by different biomass burning activities (*i.e.*, residential heating and wildfires) do not necessarily comprise the same toxicological properties.

**Table 5:** The regression analysis between PM constituents and intrinsic DTT activity (*N* = 6)^a^

PM constituents	*R*	*p*
EC	0.57	0.31
OC	**0.81**	**0.02**
WSOC	**0.89**	**0.09**
WIOC	**0.71**	**0.02**
NO_3_^−^	0.61	0.28
SO_4_^2−^	0.29	0.12
K^+^	**0.95**	**0.06**
PAHs	**0.74**	**0.03**
Ca	−0.50	0.06
Al	−0.54	0.02
Fe	−0.39	0.01
Zn	−0.76	0.02
Ba	−0.51	0.09
Cu	−0.56	0.06
Ti	−0.56	0.04
Mn	−0.67	0.02
Pb	−0.01	0.01
Ni	−0.47	0.16
Cr	0.09	0.00
V	−0.08	0.24

a The correlation coefficient values above 0.7 and the *p*-values below 0.1 are highlighted.

The high correlation of WSOC and DTT activity can in part be attributed to the photochemical reactions, as evident by significant loading of this PM constituent in Athens and the corresponding high oxidative potential. The WSOC level in Athens was primarily driven by SOA formation as the samples at this location were collected during summer, where peak photochemical activities are observed in this region.^83^ This is further supported by the substantially higher mass fraction of SO_4_^2−^, which was also elevated similar to the DTT values, in Athens (∼264 μg mg^−1^ PM) compared to Beirut (∼93 μg mg^−1^ PM), Milan (∼40 μg mg^−1^ PM), LA (∼39 μg mg^−1^ PM), Riyadh during dust event (∼37 μg mg^−1^ PM) and non-dust period (∼78 μg mg^−1^ PM). Sulfate correlation with DTT was significant (*p* = 0.12) which is probably due to its photochemical origin and correlation with WSOC, since sulfate is not generally regarded as a redox active PM component.^23,48^ Thus, the PM's high DTT activity in Athens, can be associated with the enhanced photochemical reactions in this city.

The heavy-trafficked city of LA showed moderate redox activity (28.10 ± 5.23 nmoles (min mg)^−1^) which is consistent with the moderate correlation of EC, a marker of vehicle exhaust emission, with observed DTT levels (*R* = 0.57), as well as with the high and significant correlation between DTT and WIOC (*R* = 0.89), with the latter species being a marker of primary OC emissions mainly from traffic.^78^ Metals, which are typically emitted from vehicular abrasion and combustion sources (*e.g.*, industrial and traffic emissions),^35,51,73^ are reported as one of the contributors to the PM oxidative potential.^59,84^ Specifically, transition metals have been shown to have a large oxidative capacity.^3,16^ However, the DTT consumption rates across the sites chosen in our study, were not correlated with the corresponding content of metals, as evident by negative correlation coefficient for nearly all metal species. This observation could be due to two prime reasons. First, the greater potency of WSOC and OC species on a per PM mass basis in generating redox activity compared to PM elemental content may have led to negative *R* values between DTT and the mass fraction of metal components. PM samples with high metals and low WSOC and OC contents had the lowest DTT responses. For example, the total metallic mass fraction in Riyadh was the highest among the studied cities during both dust (∼299 μg mg^−1^ PM) and non-dust periods (∼300 μg mg^−1^ PM), while the WSOC per mass content was the lowest, resulting in lowest intrinsic DTT values during dust (9.32 ± 0.80 nmoles (min mg)^−1^) and non-dust periods (12.53 ± 1.43 nmoles (min mg)^−1^) in this Middle Eastern city. Secondly, the water-solubility and oxidation state of the PM components play a major role in their oxidation activity. The consumption of the DTT assay used on the filters is primarily driven by water-soluble compounds. Therefore, the lack of correlation between DTT consumption rate and the PM total metal content, can also be attributed to the water-insoluble fraction of some metals that decreases their correlation with water-soluble DTT values.

In summary, six sets of PM samples, each dominated by unique emission sources in various locations around the globe were collected and analyzed for their content of chemical constituents and the associated oxidative potential. The DTT acellular assay was employed to quantify the redox activity of PM samples. According to our results, the highest DTT activity were observed at locations where PM were rich in OC, WSOC and PAHs, indicating that PM emitted by biomass burning activities as well as secondary organic aerosols formed by photochemical reactions have a higher oxidative potential.

## Conflicts of interest

There are no conflicts of interest to declare.

## Supplementary Material

EA-002-D2EA00043A-s001
