# Supplementary material for: The oxidative potential of particulate matter (PM) in different regions around the world and its relation to air pollution sources
Source: Environ Sci Atmos. 2022 Jul 5;2(5):1076–86. doi: 10.1039/d2ea00043a (PMC9476553; doi:10.1039/d2ea00043a)
Supplement: EA-002-D2EA00043A-s001 [file EA-002-D2EA00043A-s001.pdf]

Electronic Supplementary Material (ESI) for Environmental Science: Atmospheres.
This journal is © The Royal Society of Chemistry 2022

The oxidative potential of particulate matter (PM) in different regions around the world and its relation to air pollution sources

Vahid Jalali Farahani[1], Abdulmalik Altuwayjiri[1,6], Milad Pirhadi[2], Vishal Verma[3], Ario Alberto Ruprecht[4], Evangelia Diapouli[5], Konstantinos Eleftheriadis[5], Constantinos Sioutas[1*]

[1]University of Southern California, Department of Civil and Environmental Engineering, Los Angeles, California, USA

[2]California Air Resources Board, Sacramento, California, USA

[3]University of Illinois at Urbana Champaign, Department of Civil and Environmental Engineering, Urbana, Illinois, USA

[4]International Society of Doctors for Environment (ISDE), Italy

[5]Environmental Radioactivity Laboratory, N.C.S.R. Demokritos, 15341 Attiki, Greece

[6]Majmaah University, Department of Civil and Environmental Engineering, Majmaah, Riyadh, Saudi Arabia

*Corresponding author:

Constantinos Sioutas, Sc.D.

3620 S. Vermont Ave. KAP210, Los Angeles, CA 90089

E-mail: sioutas@usc.edu

Telephone: 213-740-6134

Fax: 213-744-1426

Table S1. Comparison of PM mass fractions of selected components in the current study with the previous measurements in the literature at each location site.

	PM Mass fraction (μg/mg PM)											
**Study**	**EC**	**OC**	**NO_3^-**	**SO_4^{-2}**	**NH_4^+**	**Ca**	**Al**	**Fe**	**Zn**	**Ba**	**Cu**	**Pb**
**Athens, Greece**												
Current study	13.36	241.64	3.93	264.17	69.57	44.54	18.53	18.76	1.35	0.45	0.28	0.26
Taghvaee et al. (2019)	39.98	184.80	1.89	69.94	--	14.22	9.71	11.45	1.32	3.50	1.16	0.77
Diapouli et al. (2017)	38.81	211.59	35.34	247.50	47.62	67.29	9.48	21.34	2.91	0.38	0.62	0.48
Amato et al. (2016)	29.45	247.57	15.72	262.47	85.70	15.90	8.18	10.18	0.90	0.63	0.20	0.25
**Beirut, Lebanon**												
Current study	10.04	90.27	0.94	93.41	31.89	58.09	13.48	8.54	4.21	0.54	0.80	0.21
Borgie et al. (2016)	--	--	79.07	209.00	43.95	57.29	13.05	15.59	1.15	1.09	1.31	0.20
**Los Angeles, USA**												
Current study	23.05	241.12	179.15	56.41	46.83	19.02	7.73	7.33	0.45	0.48	0.26	0.06
Shirmohammadi et al. (2017)	30.00	230.00	--	--	--	4.09	3.85	10.41	0.45	0.59	0.58	0.05
Habre et al. (2021)	59.84	190.76	53.53	--	24.80	3.80	3.56	5.88	0.44	0.38	0.26	0.10
**Milan, Italy**												
Current study	28.05	217.76	224.75	40.29	91.05	10.75	5.42	5.97	0.62	0.21	0.24	0.29
Amato et al. (2016)	59.21	216.50	188.36	64.19	73.05	7.87	4.95	10.52	1.87	1.74	1.87	0.17
**Riyadh, Saudi Arabia**												
Current study - dust events	6.71	34.62	20.73	37.37	2.67	134.40	58.54	39.45	0.22	0.43	0.08	0.05
Current study - non-dust event	23.00	76.55	40.45	77.97	8.33	160.76	40.05	28.17	2.16	0.54	0.22	0.19
Alharbi et al. (2015)	--	--	16.32	33.29	13.79	133.66	24.24	21.99	0.31	0.48	0.35	0.02

References

Alharbi, B., Shareef, M.M. and Husain, T., 2015. Study of chemical characteristics of particulate matter concentrations in Riyadh, Saudi Arabia. Atmospheric Pollution Research, 6(1), pp.88-98.

Amato, F., Alastuey, A., Karanasiou, A., Lucarelli, F., Nava, S., Calzolai, G., Severi, M., Becagli, S., Gianelle, V.L., Colombi, C. and Alves, C., 2016. AIRUSE-LIFE+: a harmonized PM speciation and source apportionment in five southern European cities. Atmospheric Chemistry and Physics, 16(5), pp.3289-3309.

Borgie, M., Ledoux, F., Dagher, Z., Verdin, A., Cazier, F., Courcot, L., Shirali, P., Greige-Gerges, H. and Courcot, D., 2016. Chemical characteristics of PM2. 5–0.3 and PM0. 3 and consequence of a dust storm episode at an urban site in Lebanon. Atmospheric Research, 180, pp.274-286.

Diapouli, E., Manousakas, M., Vratolis, S., Vasilatou, V., Maggos, T., Saraga, D., Grigoratos, T., Argyropoulos, G., Voutsa, D., Samara, C. and Eleftheriadis, K., 2017. Evolution of air pollution source contributions over one decade, derived by PM10 and PM2. 5 source apportionment in two metropolitan urban areas in Greece. Atmospheric environment, 164, pp.416-430.

Habre, R., Girguis, M., Urman, R., Fruin, S., Lurmann, F., Shafer, M., Gorski, P., Franklin, M., McConnell, R., Avol, E., Gilliland, F., 2021. Contribution of tailpipe and non-tailpipe traffic sources to quasi-ultrafine, fine and coarse particulate matter in southern California. Journal of the Air and Waste Management Association 71, 209–230.

Shirmohammadi, F., Wang, D., Hasheminassab, S., Verma, V., Schauer, J.J., Shafer, M.M. and Sioutas, C., 2017. Oxidative potential of on-road fine particulate matter (PM2. 5) measured on major freeways of Los Angeles, CA, and a 10-year comparison with earlier roadside studies. Atmospheric Environment, 148, pp.102-114.

Taghvaee, S., Sowlat, M.H., Diapouli, E., Manousakas, M.I., Vasilatou, V., Eleftheriadis, K. and Sioutas, C., 2019. Source apportionment of the oxidative potential of fine ambient particulate matter (PM2. 5) in Athens, Greece. Science of The Total Environment, 653,

pp.1407-1416.
